# Poor self-reported sleep is associated with prolonged white matter T2 relaxation in psychotic disorders

**DOI:** 10.3389/fpsyt.2024.1456435

**Published:** 2025-01-07

**Authors:** Umit Haluk Yesilkaya, Xi Chen, Lauren Watford, Emma McCoy, Meltem Sen, Ilgin Genc, Fei Du, Dost Ongur, Cagri Yuksel

**Affiliations:** ^1^ Schizophrenia and Bipolar Disorder Program, McLean Hospital, Belmont, MA, United States; ^2^ Bakirkoy Training and Research Hospital for Psychiatry, Neurology and Neurosurgery, Istanbul, Türkiye; ^3^ Department of Psychiatry, Harvard Medical School, Boston, MA, United States

**Keywords:** sleep, white matter, T2 relaxation, schizophrenia, bipolar disorder, psychosis

## Abstract

**Background:**

Psychotic disorders are characterized by white matter (WM) abnormalities; however, their relationship with the various aspects of illness presentation remains unclear. Sleep disturbances are common in psychosis, and emerging evidence suggests that sleep plays a critical role in WM physiology. Therefore, it is plausible that sleep disturbances are associated with impaired WM integrity in these disorders. To test this hypothesis, we examined the association of self-reported sleep disturbances with WM transverse (T2) relaxation times in a cross-diagnostic sample of patients with psychosis.

**Methods:**

A total of 28 patients with psychosis (11 schizophrenia spectrum disorders and 17 bipolar disorder with psychotic features) were included. Metabolite (N-acetyl aspartate, choline, and creatine) and water T2 relaxation times were measured in the anterior corona radiata at 4T. Sleep was evaluated using the Pittsburgh Sleep Quality Index (PSQI).

**Results:**

PSQI total score showed a moderate to strong positive correlation with water T2 (*r* = 0.64, *p*< 0.001). Linear regressions showed that this association was independent of the overall severity of depressive, manic, or psychotic symptoms. In our exploratory analysis, sleep disturbance was correlated with free water percentage, suggesting that increased extracellular water may be a mechanism underlying the association of disturbed sleep and prolonged water T2 relaxation.

**Conclusion:**

Our results highlight the connection between poor sleep and WM abnormalities in psychotic disorders. Future research using objective sleep measures and neuroimaging techniques suitable to probe free water is needed to further our insight into this relationship.

## Introductıon

An expanding body of literature indicates disrupted white matter (WM) microstructure in schizophrenia (SZ) (1), including in medication-free first-episode (FE) patients (2) and unaffected relatives (3). Bipolar disorder (BD), which also frequently presents with psychosis (4), exhibits similar WM abnormalities (5, 6). The majority of evidence for WM pathology is derived from diffusion tensor imaging (DTI) studies, and the most commonly reported measure, fractional anisotropy (FA), does not provide information about the specific biological components affected (7). Nonetheless, additional lines of evidence, including other DTI measures, novel imaging techniques, and post-mortem and genetic studies, suggest alterations in several aspects of WM microstructure, including axon, myelin, and extracellular water (1, 8–10). However, the link between WM abnormalities and illness presentation in psychotic disorders is not clear, as attempts to identify symptom correlates have largely been unfruitful.

Recent evidence suggests that sleep disturbances are associated with disrupted WM microstructure. In healthy individuals, poor sleep was associated with altered FA and other diffusivity measures in the whole brain and specific WM tracts (11–19), and sleep deprivation was associated with widespread alterations in WM microstructure (20, 21). In addition, in primary insomnia disorder, FA was reduced in the internal capsule (22, 23), thalamus–pars triangularis tracts (24), and several regions, including the internal capsule, corona radiata, longitudinal fasciculus, and corpus callosum (25).

Sleep disturbances are highly prevalent in psychotic disorders and are present even when patients are clinically stable or in the euthymic state (26, 27). However, despite the accumulating evidence indicating a role for sleep in WM physiology, little is known about the link between sleep disturbances and the WM disruptions observed in these disorders. To the best of our knowledge, there are no studies in SZ that examined this relationship. In a recent study in individuals at ultrahigh risk for psychosis, poor sleep was associated with lower FA in the corpus callosum, and both increased and decreased FA in the ventral brain regions (28). In BD, lower objective and self-reported sleep duration correlated with reduced FA and increased radial diffusivity (RD) in multiple WM tracts (Benedetti et al., 2017). In contrast, in another study, poor sleep (reduced sleep duration and more sleep inertia) was associated with higher FA in several WM tracts (Verkooijen et al., 2017).

Transverse relaxation time (T2) refers to the duration required for the decay of magnetization in the transverse plane. It reflects the spin–spin interactions and is affected by the homogeneity of the molecular environment and molecular motion. It is longer when nuclei are in free motion and in an environment where interaction with other types of magnetic nuclei is relatively limited, such as in cerebrospinal fluid (29). T2 can be measured for water and intracellular metabolites such as N-acetyl aspartate (NAA), choline (Cho), and creatine (Cr). Water T2 can provide information on WM macromolecule structure and fluid homeostasis, while the metabolite relaxation times reflect the intra-axonal milieu. In a previous study, we observed increased water T2 as well as a reduced NAA T2 in chronic SZ compared to controls (30), suggesting an impoverishment of WM macromolecule structures and abnormal intra-axonal milieu and volume. Prolonged WM water T2 in SZ has also been reported in previous studies (31–33). We also observed that NAA T2 is significantly reduced in the chronic psychosis compared to FE subjects, suggesting that apparent NAA concentration reductions reported in psychotic disorders may indeed reflect shortened T2 and not lower NAA tissue concentration (34). More recently, in a longitudinal study in FE psychosis, we observed a significant reduction of NAA in the second year of the follow-up compared to baseline, while the water T2 showed a trend of increase (35).

Given this background, we hypothesized that sleep disturbances would be associated with altered WM T2 in psychosis. To test this hypothesis, we examined the link between self-reported sleep quality and WM water and metabolite T2 in a cross-diagnostic sample of patients with psychotic disorders [SZ spectrum disorders (SSD) and BD with psychotic features (BDP)]. Sleep supports neuronal integrity and neuroplasticity, as well as myelin physiology and brain fluid homeostasis. Therefore, we hypothesized that poor sleep quality would be associated with alterations in both metabolite and water T2.

## Materials and methods

### Participants

This is a secondary analysis of data obtained in two studies performed at McLean Hospital, which acquired T2 data with identical protocols on the same magnetic resonance imaging (MRI) scanner. One of these studies was a longitudinal neuroimaging study in FE psychosis. The other study was a multimodal neuroimaging, genetic, and metabolic study in SZ and BD. Patients were recruited from the inpatient and outpatient services at McLean Hospital. Participants with any uncontrolled medical disorders, intellectual disability, neurological sequela, history of head trauma with loss of consciousness, and contraindication to MRI were excluded. The studies were approved by McLean Hospital and Mass General Brigham institutional review boards, and all participants provided written informed consent. The study procedures adhered to the principles outlined in the Declaration of Helsinki. A total of 28 patients (17 BDP and 11 SSD) provided information about sleep quality within 1 month of their scan (average interval 11.4 ± 10.6 days) and were included in this study. The SSD group consisted of nine individuals with schizoaffective disorder (SZA) and two individuals with psychotic disorders, not otherwise specified (Psy-NOS). The sample consisted of predominantly early-course patients, with 80.8% of the patients within the first 3 years of illness onset. Demographic and clinical information in this sample is displayed in **Table 1**.

**Table 1:** Clinical and demographic variables and T2 measures in the sample.

	Whole sample (*n* = 28)	BDP (*n* = 17)	SSD (*n* = 11)
Age	24.3 ± 4.9 (19–44)	23.9 ± 3.2 (19–29)	24.9 ± 6.8 (19–44)
Sex (% female)	35.7%	41.2%	27.3%
BMI	24.9 ± 4.6 (18.6–34.7)	24.3 ± 4.4 (18.6–33.9)	25.8 ± 4.8 (19.8–34.7)
Education and employment status			
In school	25%	23.5%	27.3%
Working	39.3%	47.1%	27.3%
Unemployed	35.7%	29.4%	45.5%
Diagnosis and history			
SZA	32.1%	N/A	N/A
Psy-NOS	7.1%	N/A	N/A
BDP	60.7%	N/A	N/A
Duration of illness (years)	2.8 ± 2.5 (0–11)	2.9 ± 2.9 (1–11)	2.6 ± 1.4 (0–5)
Medications			
Any medication	85.7%	82.4%	90.9%
Antipsychotics	67.9%	58.8%	81.8%
CPZ	225.9 ± 226.9 (0–800)	191.2 ± 207.8 (0–600)	285 ± 256.4 (0–800)
Lithium	42.9%	47.1%	36.4%
Other mood stabilizers	28.6%	41.2%	9.1%
Sleep medications	3.5%	0%	9.1%
Symptom severity			
YMRS	6.5 ± 8.1 (0–36)	4.1 ± 4.9 (0–21)	10.3 ± 10.6 (0–36)
MADRS	9.2 ± 8.3 (0–28)	6.8 ± 7.8 (0–27)	12.8 ± 8.2 (4–28)
PANSS total	45.4 ± 13.3 (30–76)	37.9 ± 8.6 (30–57)	56.9 ± 10.9 (36–76)
PSQI total	5.6 ± 2.5 (1–11)	6.2 ± 2.7 (1–11)	4.8 ± 2 (1–7)
T2 (ms)			
Water	64 ± 3.4 (57.9–70.6)	64.7 ± 4 (57.9–70.6)	62.9 ± 1.9 (60.1–65.8)
NAA	255.7 ± 23.8 (202.8–302.3)	254.6 ± 27.4 (202.8–302.3)	257.3 ± 18.4 (208–274.3)
Cr	148 ± 12 (123.0–173.6)	148.1 ± 12.3 (123.0–165.7)	147.8 ± 12.2 (129.1–173.6)
Cho	175.5 ± 22 (137.5–209.9)	180.8 ± 23 (137.5–209.9)	167.3 ± 18.3 (143–201.7)

Some of the data are presented as mean ± standard deviation (minimum–maximum). “Any medication” refers to users of any psychiatric medications. YMRS and MADRS scores reflect the totals with sleep items included. BDP, bipolar disorder with psychotic features; BMI, body mass index; CPZ, chlorpromazine equivalent of antipsychotic dose; MADRS, Montgomery-Asberg Depression Rating Scale; PANSS, Positive and Negative Syndrome Scale; PSY-NOS, psychotic disorder, not otherwise specified; SSD, schizophrenia spectrum disorders (SZA and Psy-NOS); SZA, schizoaffective disorder; YMRS, Young Mania Rating Scale.

N/A, not applicable.

### Clinical assessments

Sleep was assessed using the Pittsburgh Sleep Quality Index (PSQI) (36), a self-report questionnaire that probes the sleep quality and disturbances over a 1-month period. The composite score, PSQI total score, was used in all analyses. A higher PSQI total score reflected poorer sleep. Diagnoses were ascertained using the Structured Clinical Interview for DSM-IV (SCID). In addition to PSQI, the severity of psychotic, manic, and depressive symptoms was assessed using the Positive and Negative Syndrome Scale (PANSS), the Young Mania Rating Scale (YMRS), and the Montgomery-Asberg Depression Rating Scale (MADRS). Antipsychotic load was calculated as the total chlorpromazine equivalent dose (CPZ) (37).

### MRI

**Figure 1:**
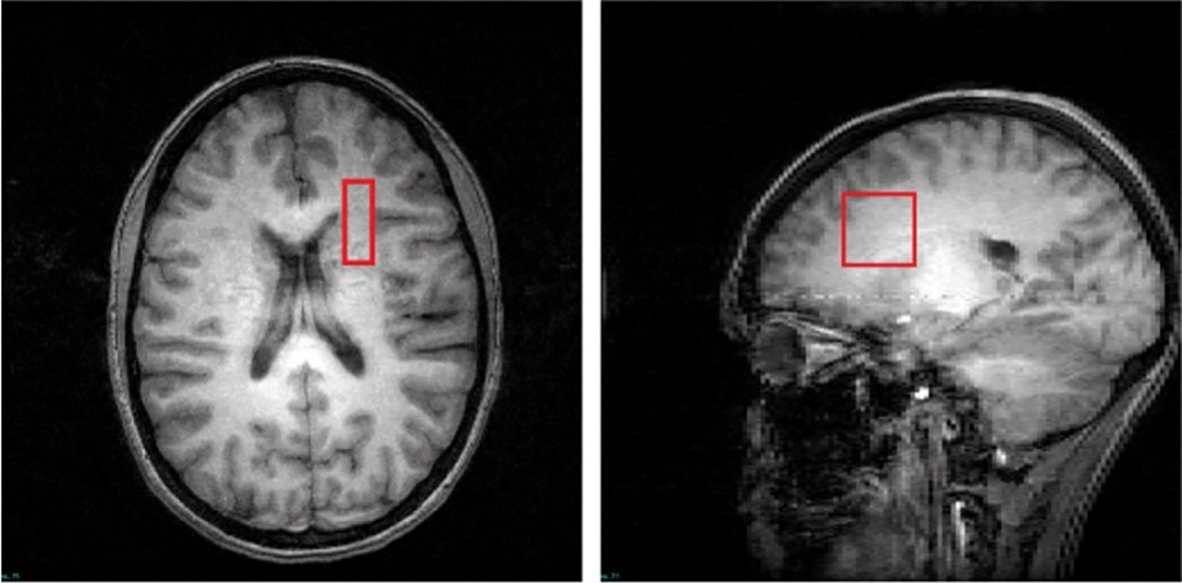
T1-weighted images in the transverse and sagittal planes depict the voxel placement.

T2 relaxation time measurements were conducted on a 4T Varian full-body MR scanner (Unity/Inova; Varian NMR Instruments, CA, USA) using a 16-rung, single-tuned, volumetric birdcage coil. Global shimming was performed, followed by the acquisition of high-contrast T1-weighted sagittal images, which served to position the axial images and MRS voxels. A 1 × 3 × 3 cm^3^ single MRS voxel (**Figure 1**) was then placed on the corona radiata, centered at the level of the genu of the corpus callosum but lateral to it (i.e., it does not include any callosal fibers). Anterior corona radiata was chosen because previous studies reported altered T2 relaxation in the prefrontal cortex (PFC) in SZ (Williamson et al., 1992), and this particular region allowed the placement of a large enough WM voxel—underlying the PFC—that ensured a high signal-to-noise ratio. The voxel was placed in pure WM with adjacent gray matter in the anterior and lateral directions used as anchors to ensure that the location was consistent across scans. SPM12 was used for tissue segmentation on the T1. The MRS voxel tissue percentages were calculated using AFNI, and the voxel was consistently positioned in WM mostly (88% ± 4% of WM percentage). Localized shimming was performed to ensure water linewidths< 15 Hz.

Water and metabolite (NAA, Cr, and Cho) T2 spectra were obtained using a PRESS sequence modified with four varying TEs (30, 90, 120, and 200 ms) and TR = 3,000 ms; 48 repetitions for metabolite and 8 repetitions for water T2 relaxation time measurements. A 3-ms sinc pulse with a bandwidth of 2,000 Hz was used for excitation; two 6-ms Varian optc4 (Optimized Control Pulse for 4 zero sinc pulse) pulses with a bandwidth of 1,050 Hz were used for refocusing.

We measured magnetizations *M(t)* of water and each metabolite with *t* = 30, 90, 120, and 200 ms and fitted their T2s using the mono-exponential fitting function:


$$M\left(t\right)=M_{0}\cdot \text{exp}\left(-t/T2\right)$$

We further performed a bi-exponential fitting for water T2 relaxometry to investigate the water compartments, as we found that the mono-exponential fitting may not fully account for water T2 decay (**Supplementary Figure S1**). The bi-exponential fitting function is:


$$M\left(t\right)=M_{fast}\cdot \text{exp}\left(-t/T2_{fast}\right)+M_{slow}\cdot \text{exp}\left(-t/T2_{slow}\right)$$

where T2_fast (<80 ms) reflects intra- and extracellular water relaxation, while T2_slow (>120 ms) can be considered as free water relaxation. The free water percentage (FW%) can also be calculated: *FW*% = *M_slow_*/(*M_slow_* +*M_fast_*).

### Statistical analyses

Statistical analyses were performed using IBM SPSS Statistics Version 26. Pearson or Spearman’s rank correlation were used to examine the correlations between sleep disturbance and T2 relaxation, depending on the distribution (normal vs. non-normal) and the type of (continuous vs. ordinal) data.

Linear regressions were used to test the associations of PSQI total score with neuroimaging measures of interest adjusted for demographic and clinical variables. Partial regression plots and a plot of studentized residuals against the predicted values indicated that assumptions of linear relationship were met. A histogram and a P–P plot of standardized residuals showed a normal distribution. Variance inflation factors (VIFs) indicated that no confounding multicollinearity was present.

All analyses were two-tailed. The significance level for hypothesis testing (α) was set at 0.05. For exploratory analyses, the Benjamini–Hochberg procedure was used to correct for multiple comparisons with the threshold for false discovery rate (FDR) at 0.05, and adjusted *p*-values are presented.

## Results

**Figure 2:**
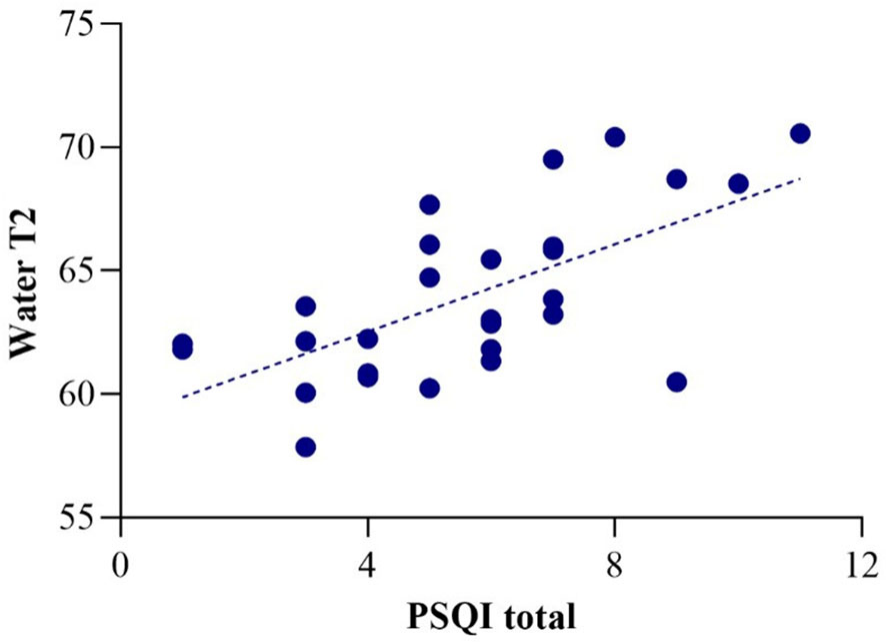
Correlation of water T2 with PSQI total score (*r* = 0.64).

In the whole patient sample, the average PSQI total score was 5.64, above the cutoff value of 5, which indicates “poor sleep” (36). PSQI total score was positively correlated with water T2 (*r* = 0.64, *p* = 10^-4^ × 2; **Figure 2**). There were no other significant correlations with the PSQI total score and any of the metabolite T2 values in the whole sample (all *p* > 0.5). Among the PSQI component scores (36), water T2 was significantly correlated with sleep disturbance and latency (Spearman’s rho = 0.63, *p*< 0.001 and Spearman’s rho = 0.41, *p* = 0.029, respectively).

There were no differences in any sociodemographic (age and sex) and clinical (BMI, duration of illness, antipsychotic dose, and symptom scale scores) variables between SSD and BDP, except higher PANSS total and YMRS scores in SSD [*t*(26) = 5.14, *p*< 0.001 and *t*(26) = 2.09, *p* = 0.047, respectively]. PSQI total and component scores, as well as water and metabolite T2 values, were also not different between these groups (all *p* > 0.05). SSD and BDP displayed almost identical correlations between PSQI total and water T2 (*r* = 0.63, *p* = 0.046 and *r* = 0.63, *p* = 0.013).

We explored whether the association of sleep disturbance with water T2 was specific to this symptom dimension or simply a by-product of increased severity of manic, depressive, or psychotic symptoms. For this purpose, separate linear regressions were carried out with water T2 as the dependent variable, and with YMRS, MADRS, or PANSS total scores included as predictors in addition to the PSQI total score. Sleep item scores were subtracted from YMRS and MADRS total scores to isolate the contribution of sleep disturbance by PSQI total score. Age and sex were also included as additional covariates in all analyses. These analyses showed that sleep disturbance (PSQI total score) remained a significant predictor of water T2 in the whole sample, independent of manic, depressive, or psychotic symptom severity (β = 0.62, *p* = 0.004, β = 0.62, *p* = 0.005, and β = 0.62, *p* = 0.008, respectively).

We also explored whether the association of sleep disturbance with water T2 was independent of duration of illness and antipsychotic dose. Separate linear regressions with water T2 as the dependent variable included duration of illness or CPZ as predictors in addition to PSQI total score. Age and sex were included as additional covariates. These analyses also showed that PSQI total remained a significant predictor of water T2 in the whole sample, independent of duration of illness and antipsychotic dose (β = 0.38, *p* = 0.043 and β = 0.63, *p* = 0.005, respectively).

In further exploratory analysis, we found that the mono-exponential fitting quality was inversely correlated with PSQI total score (spearman’s rho = −0.52, *p* = 0.009). The association between mono-exponential fitting quality and PSQI score implies that additional compartments of water may affect the mono-exponential fitting quality and thus could be a biomarker of WM integrity related to sleep quality. Therefore, we performed analyses with bi-exponential fitting for T2 decay, and found that PSQI total score was positively correlated with FW% (spearman’s rho = 0.42, *p* = 0.032) (**Supplementary Figure S2**) but not with T2_fast (*r* = 0.08, *p* = 0.666), suggesting that increased free water may be a potential mechanism underlying the association of poor sleep with prolonged water T2.

## Discussion

In this study, we examined the association of sleep quality with prefrontal WM water and metabolite T2 in patients with BDP and SSD. Supporting our hypothesis, we found that self-reported poor sleep was associated with prolonged water T2. This association was independent of the severity of other manic, depressive, or psychotic symptoms and other clinical factors including duration of illness and antipsychotic medication dose.

Water T2 reflects the interaction of water with nonaqueous molecules in its microenvironment and is prolonged in conditions where the frequency of these interactions is reduced due to the relative expansion of the water component (38). Prolonged WM water T2 has been reported in SZ (30, 33, 39), with suggestions that such findings may arise from disruptions in myelin integrity, reduced axon size, or increased interstitial fluid. If altered water T2 in SZ indeed originates from myelin abnormalities, the correlation of poor sleep with longer water T2 in our sample would be consistent with the expanding literature, which indicates that sleep influences oligodendrocyte function, expression of numerous genes related to membrane metabolism and myelination, and sleep deprivation leads to myelin disruption (reviewed in 40). Furthermore, sleep is crucial for neuronal homeostasis and neuroplasticity (41–43), with poor sleep linked to reduced gray matter in the PFC (44–49), a region where the anterior corona radiata fibers project to. In addition, abnormal sleep and experimental sleep deprivation lead to impairments in executive functions, which are mediated by the PFC (45, 48–50) and are associated with the corona radiata microstructure (51, 52). Consequently, it is plausible that sleep disruption-related neuronal alterations in the PFC are accompanied by reduced axon size in the corona radiata, thereby manifesting as a relative expansion of the water component. However, the lack of correlation between sleep disturbance and metabolite T2 in our sample argues against this possibility, as a decrease in axon size would be expected to prolong the T2 relaxation for intracellular metabolites.

The positive correlation of FW% with PSQI total score suggests that increased FW due to poor sleep is another potential mechanism, which would be consistent with a recent study (19), although our cross-sectional study cannot provide information about any causal relationship. An increase in FW has been documented in the early course SZ patients (53–58) and in individuals at clinical high risk for psychosis (59), and has been shown to be inversely correlated with the duration of illness (58). Our sample consisted predominantly of early-course patients, which also supports this possibility. Other than free water imaging based on DTI (60, 61), multi-exponential T2 relaxometry has been a potentially useful technique for characterizing the tissue water compartments (62, 63). The compartment with the shortest T2 (10–20 ms) is usually regarded as myelin water (64, 65), and it can hardly be detected with the current T2 spectroscopy protocol as our shortest TE is 30 ms. The main water compartment observed by the current study is T2 = 40–80 ms, which is mainly contributed by intra- and extracellular water (66). The T2 values we obtained from the mono-exponential fitting mostly reflected the T2 decay of this compartment. Another water compartment observed by the bi-exponential fitting is with T2 > 120 ms and is usually regarded as free water (66, 67). It has a much lower fraction compared to intra- and extracellular water, while it brings long tails to the T2 decay curves (68) and deviations from the mono-exponential fitting. It should be noted that the T2s of this compartment (T2_slow) in the current study are<400 ms and thus are not contributed by CSF, which has a very long T2 from 800 to 3,000 ms and minimal tissue percentages of the MRS voxel in the current study (0.1% ± 0.1%). Neuroinflammation has been proposed as a potential mechanism that leads to increased FW in SZ (58), and consistent with this hypothesis, previous investigations showed that increased peripheral levels of pro-inflammatory cytokines in SZ are associated with the expansion of the FW compartment (69–71). Notably, there is substantial evidence indicating that sleep disturbance and duration are linked to systemic inflammation (72, 73). Finally, recent evidence suggests that brain fluid dynamics are tightly linked to sleep–wake states, with increased cerebrospinal fluid (CSF) influx and significant augmentation of interstitial fluid observed during sleep (74). Although this burgeoning area of research has not been explored in SZ or BD, it is conceivable that abnormal CSF or glymphatic system dynamics due to poor sleep contribute to the observed increase in FW. Given the cross-sectional design of our study, we cannot speculate on the causal directionality of these potential mechanisms.

Our study had several limitations. First, given the constraints on experiment time, we have acquired signals of only four different TEs to measure T2. With limited data points, the exploratory bi-exponential analysis could be subject to inaccuracy and is not able to address the contribution from the ultra-short T2 components such as myelin. On the other hand, we acquired the full FID signal of each TE from a well-shimmed MRS voxel instead of just a few signal points using fast multi-echo imaging to achieve better signal reliability. The Carr–Purcell–Meiboom–Gill (CPMG) method used in multi-echo imaging is also sensitive to inhomogeneous B1 (RF) and B0 (static) fields (65, 67). Despite the limited number of TEs, our bi-exponential fitting showed significant improvement compared to mono-exponential fitting (**Supplementary Figure S1**). Nonetheless, given this limitation, the correlation of poor sleep with FW% should be treated as a preliminary finding offering potential guidance for future investigations exploring this relationship using suitable neuroimaging techniques. Second, because of the relatively small sample size and to avoid inflating the error rate, we could not take into account other potential factors that could affect WM T2, such as body mass index. However, in previous studies (30, 35), we did not find any association of WM water T2 with demographic or clinical factors except for sex, which is already controlled for in the regressions. Third, we did not have any objective sleep data. Therefore, we could not explore the convergence with the self-reported sleep disturbances. Finally, while all the participants were psychotic, our sample consisted mostly of patients with affective symptoms (SZA and BD). Therefore, future studies should investigate if similar associations are present in “non-affective” psychosis.

In this study, our aim was to investigate the association between sleep disturbances and WM characteristics in individuals with psychotic disorders. To this end, we deliberately adopted a cross-diagnostic approach rather than restricting our analyses to a single diagnosis, given the substantial overlap in clinical presentations, neurobiology, and genetic backgrounds between SSD and BD (75, 76). This methodology aligns with previous research by our group (34, 35, 77–83) and others (53, 84–87), and it enhances the generalizability of our findings across psychotic disorders. Importantly, there is currently no evidence suggesting distinct patterns in the association between sleep disturbances and WM biology among these disorders. Indeed, despite small sample sizes, we observed an identical correlation between PSQI total scores and water T2 relaxation rates in both diagnostic groups (Pearson’s *r* = 0.63; see Results). Given the limited research on this topic, our study provides a starting point for future investigations with larger samples that can explore potential differences among specific diagnoses. Sleep disturbances in psychotic disorders can be illness-related as well as due to educational and occupational demands. The association between sleep disturbances and prolonged T2 relaxation times may not be exclusive to psychotic disorders but can also be observed in other conditions characterized by WM abnormalities. Undiagnosed sleep apnea may have contributed to the association we observed between sleep disruption and water T2 relaxation times (88). Studies employing polysomnography can probe the contribution of this factor.

In conclusion, our findings suggest that poor sleep quality is associated with WM abnormalities in patients with psychotic disorders. Increased free water, possibly due to neuroinflammation, is a possible mechanism underlying this association. Future studies should include additional objective sleep measures and specialized neuroimaging techniques that probe the free water component in the WM.

## Data Availability

Study protocols used to generate the data restrict sharing them with outside parties. Requests to access the datasets should be directed to ayuksel@mgb.org.
