# Supplementary material for: Poor self-reported sleep is associated with prolonged white matter T2 relaxation in psychotic disorders
Source: Front Psychiatry. 2025 Jan 7;15:1456435. doi: 10.3389/fpsyt.2024.1456435 (PMC11747379; doi:10.3389/fpsyt.2024.1456435)
Supplement: Supplementary file 1 [file DataSheet1.docx]

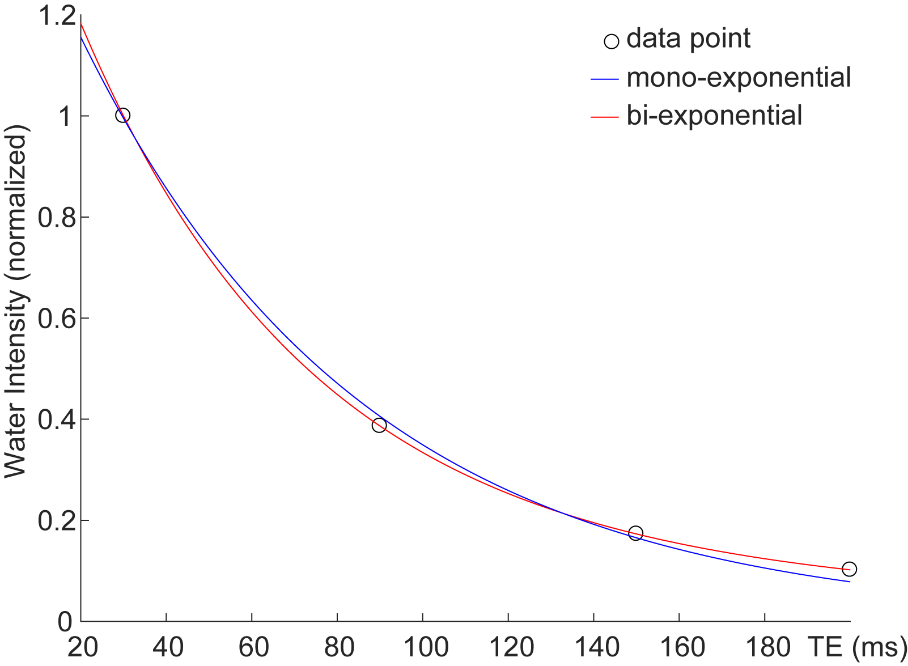


**Figure S1.** A representative T2 relaxation data set with mono-exponential and bi-exponential fittings


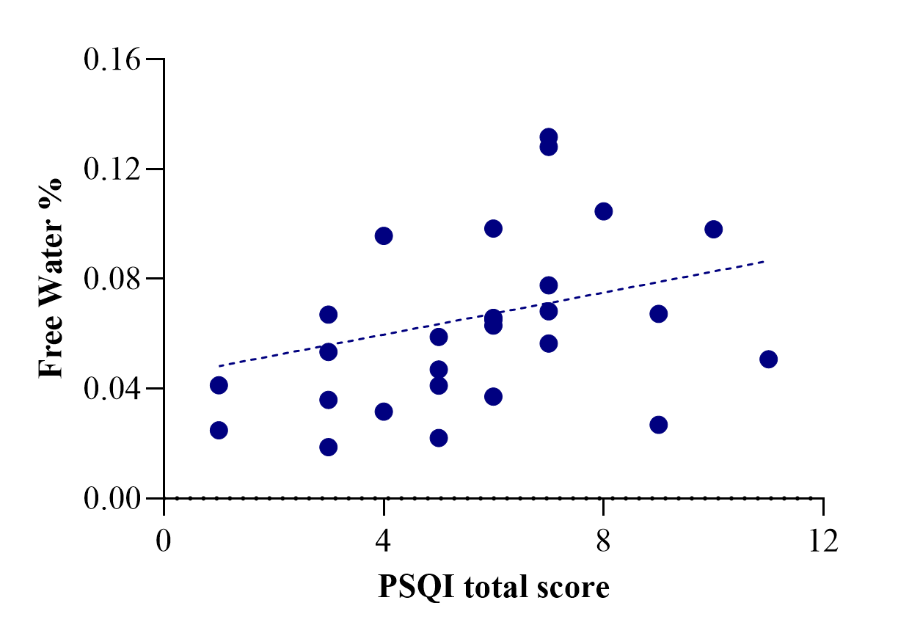


**Figure S2.** Correlation of PSQI total score with free water %.
